# Comparative Study on Phenolic Compounds and Antioxidant Activities of Hop (*Humulus lupulus* L.) Strobile Extracts

**DOI:** 10.3390/plants11010135

**Published:** 2022-01-04

**Authors:** Jae Il Lyu, Jaihyunk Ryu, Kyoung-Sun Seo, Kyung-Yun Kang, Sang Hoon Park, Tae Hyun Ha, Joon-Woo Ahn, Si-Yong Kang

**Affiliations:** 1 Department of Horticulture, College of Industrial Sciences, Kongju National University, Yesan 32439, Korea; jaeil@kongju.ac.kr (J.I.L.); hath1002@naver.com (T.H.H.); 2 Advanced Radiation Technology Institute, Korea Atomic Energy Research Institute, Jeongeup-si 56212, Korea; jhryu@kaeri.re.kr (J.R.); joon@kaeri.re.kr (J.-W.A.); 3 Jangheung Research Institute for Mushroom Industry, Jangheung 59338, Korea; astragali@daum.net; 4 Suncheon Research Center for Natural Medicines, Suncheon 57922, Korea; nms-kang@nate.com; 5 Hop&Hope Agricultural Co., Ltd., Buan 56319, Korea; shakes4u@naver.com

**Keywords:** hop, phenolic compound, antioxidant activity, DPPH, ABTS

## Abstract

In this study, we investigated the phenolic compounds in hop strobile extracts and evaluated their antioxidant property using DPPH and ABTS assay. The total phenolic compound (TPC) and total flavonoid compound (TFC) estimated in two different solvent extracts considerably varied depending on the extraction solvent. The most abundant phenolic compound in hop strobile was humulones (α-acid) with levels ranging from 50.44 to 193.25 µg/g. El Dorado accession revealed higher antioxidant activity in ethanol extracts (DPPH: IC_50_ 124.3 µg/mL; ABTS: IC_50_ 95.4 µg/mL) when compared with that of the other accessions. Correlations between DPPH (IC_50_) scavenging TFC in ethanol extract (TFC_E, −0.941), and TPC_E (−0.901), and between ABTS (IC_50_) scavenging TFC_E (−0.853), and TPC_E (−0.826), were statistically significant at *p* < 0.01 level, whereas no significant correlation was observed between antioxidant activities, TPC and TFC in water extract. This study is the first to report that variations in the level of phenolic contents and antioxidant activity of various hop cultivars depended on the type of extraction solvent used and the cultivation regions. These results could provide valuable information on developing hop products.

## 1. Introduction

Hop (*Humulus lupulus* Linnaeus), a perennial plant belonging to the Cannabaceae family, has become a widely grown agricultural plant because it is used for providing bitterness and aroma to beer [1]. Hop originated in Europe and west Asia, and are cultivated in the United States, Germany, Czech Republic, and England [2]. Historically, the flower extracts of hop, commonly known as hops, have been used in traditional medicine for treating human health because of their sedative, anti-inflammatory, antiseptic, and antidiuretic properties [3]. Various therapeutic effects of hop have increased its interest as a potential bioactive source in the pharmaceutical industry. In Korea, until the 1980s, hop was sufficiently cultivated to meet the demand; however, at present, Korea is dependent on imported hops. However, the Korean craft beer market has been growing in recent years, leading to increased interest toward specialty hops that are produced using domestic hop cultivars.

Hops are a dioecious species and unfertilized female inflorescence are commonly called cones (or strobiles). These cones are rich in unique phenolic compounds such as prenylated flavonoids, humulones (α-acids) and lupulones (β-acids) [3]. The prenylated flavonoids that include xanthohumol and 6/or 8-prenylnaringenin, act as positive modulators of GABA-induced responses in GABA receptors [4] and have been characterized as cancer chemopreventive agents [5]. The alpha acids contain main components such as humulone, cohumulone, and adhumulone, wherein pre-/post-humulone are present in low concentrations. The beta acids are one of the main constituents of the hops resin and also consist of five different components such as lupulone, colupulone, adlupulone, and pre-/post-lupulone [6]. Humulones and lupulones, two of the most abundant phenolic compounds in hops, are considered as major hop antioxidants.

The antioxidant activity of hop or hop extracts was extensively evaluated using different assays, such as the 2,2′-azino-bis(3-ethylbenzthiazoline-6-sulfonic acid) (ABTS) hydroxyl radical scavenging, 2,2-diphenyl-1-picryl-hydrazyl-hydrate (DPPH) free radical, and ferric reducing antioxidant power (FRAP) assays [7,8,9,10]. Previous literatures have consistently reported the correlation between antioxidant activity and different phenolic contents in hop. In particular, the total phenolic content (TPC) and total flavonoid content (TFC) significantly contributed to the antioxidant activity. However, only few hop cultivars or an industrial extract form that includes plug and pellet have been investigated. Recently, new hop cultivars, such as Calypso (released in 2003, USA) and El Dorado (released in 2010, USA) have been released, and research on the antioxidant activity of these cultivars is currently lacking.

Thus, the aim of the present study was to determine the composition of phenolic compounds in the extracts of hop strobile from six hop cultivars using LC-MS analysis, and to assess their antioxidant activity by ABTS and DPPH assay. Moreover, the levels of antioxidant activity and composition of the phenolic compounds changed depending on their cultivated environments or the type of extraction solvents used. Thus, we additionally (i) evaluated the changes in the phenolic compounds and antioxidant activity according to their cultivated environments, which was investigated using Saaz cultivar planted in four different regions; and (ii) confirmed the changes in the level using different extraction solvent, wherein we used 60% EtOH and water mixture for detecting antioxidant activity and measuring the TPC and TFC levels.

## 2. Results and Discussion

### 2.1. TPC and TFC Contents

**Figure 1:**
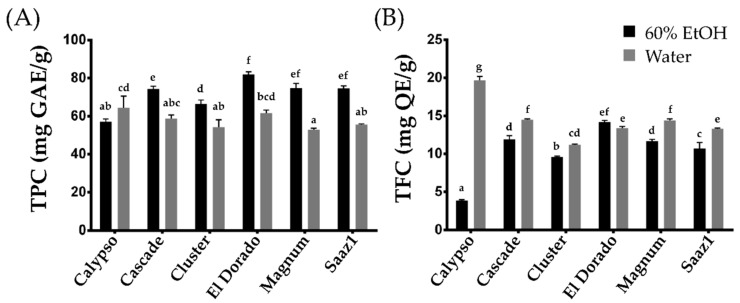
Total Phenolic and total flavonoid content of hop accessions. (**A**) Total Phenolic content, (**B**) Total flavonoid content. Letters above each point indicate a significant difference at the 5% level (Tukey HSD tests, *n* = 3).

The TPC and TFC were extracted by two different extraction methods, which included 60% ethanol and boiled-water extracts. The efficiency of the phenolic compounds was influenced by several critical factors, such as the extraction solvent, temperature, time, and pH, wherein their impact can either be independent or interactive [11]. In the present study, the levels of TPC extracted by ethanol (TPC_E) and that extracted by water (TPC_W) ranged from 57.00 (Calypso) to 81.90 (El Dorado) mg GAE g^−1^, and 52.80 (Magnum) to 64.50 (Calypso) mg GAE g^−1^, respectively (Figure 1A). The TPC levels in water extracts decreased in all cultivars except Calypso, which observed an increase in the levels of TPC. In contrast, under the same extraction methods, the TFC levels ranged from 3.90 (Calypso) to 14.20 (El Dorado) mg QE g^−1^ (TFC_E), and from 11.20 (Cluster) to 19.70 (Calypso) mg QE g^−1^ (TFC_W) (Figure 1A). Considering the yield, the TPC content tends to increase in the ethanol extract, whereas the TFC levels increased in the water extracts. In particular, the TFC levels in the water extract of the Calypso cultivar increased approximately four times more than that in the ethanol extract, indicating that the cultivar is rich in water-soluble phenolic compounds. This cultivar can be a valuable genetic resource to further study the changes in TFC characteristics between aqueous and ethanol extracts in hop.

A previous study between the aqueous and hydroalcoholic hop extracts also observed similar results for the TPC levels. Kowalczyk et al. [12] reported that the TPC and TFC levels in hydroalcoholic extracts containing 50% methanol and 50% ethanol were significantly higher than that of the aqueous extract. In our study, the average level of TPC_E at 71.53 mg GAE g^−^^1^ and that of TPC_W at 57.88 mg GAE g^−1^ were consistent with the previous results, but the TFC content in both extracts either showed similar levels (El Dorado) or increased levels in water extract.

**Figure 2:**
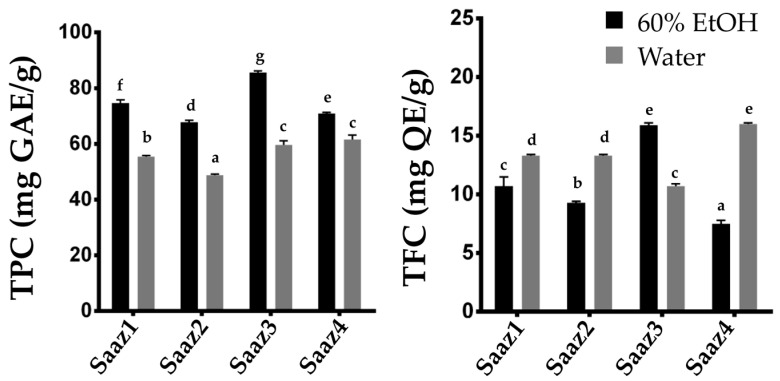
Comparison of TPC and TFC contents of Saaz cultivar from different cultivated regions. Total Phenolic content (**left**), Total flavonoid content (**right**). The letters above each point indicate a significant difference at the 5% level (Tukey HSD tests, *n* = 3).

Additionally, the level of phenolic contents varied depending on their species or cultivars and the environmental condition as described in previous literatures [13]. The TPC content in hop strobile extracts, widely ranged from 8.7 to 26.5 μg GAE g^−1^ [14], from 44.22 to 54.78 mg GAE g^−1^ [12], and 887 mg g^−1^ [15]. Therefore, we investigated changing levels of phenolic compounds by estimating their content in hops cultivated in different regions (Figure 2). The Saaz cultivar from Czech, one of the famous hop varieties, is widely cultivated in Korea because of its excellent adaptability to the environmental conditions. As expected, the TPC and TFC levels significantly varied in the ethanol extracts depending on their cultivated regions. Overall, the phenolic compounds in hop strobiles differed depending on the cultivars, extraction solvents, and cultivated regions, which is consistent with the results of previous studies.

### 2.2. Analysis of Prenylflavonoids and Hop Acids

Table 1 shows the identification of prenylflavonoids and hop acids by LC-MS in hop strobile extracts. First, the major hop acids observed in six hop cultivars were identified as: humulones, colupulone, and lupulone, which ranged from 50.44 (Magnum) to 193.25 µg/g (Cascade), from 35.37 (Saaz1) to 73.00 µg/g (Cascade), and from 17.41 (El Dorado) to 52.10 µg/g (Calypso), respectively. In contrast, adlupulone and 6-prenylnaringenin (6-PN) were present in relatively small amounts, especially 6-PN, which remained undetected in the three cultivars Saaz1, Cluster, and El Dorado (Table 2). Cascade showed higher levels of 6-prenylnaringenin (29.78 µg/g), cohumulone (77.87 µg/g), and colupulone (73.0 µg/g). Although collected from different regions, Saaz2 exhibited higher levels of xanthohumol (53.6 µg/g), humulones (193.25 µg/g), and adlupulone (8.47 µg/g) compared to that of the other cultivars. Humulones and lupulones, which are well-known major flavonoids in hop, are considered as principal bioactive compounds based on their biological and pharmacological properties, such as sedative and cancer chemopreventive activities, antimicrobial activity against bacteria, fungi and viruses, and antioxidant activity [16,17,18]. Prenylated flavonoids are found in a few families including *Cannabaceae*, *Moraceae*, *Rutaceae,* and *Umbelliferae* [19], and these compounds also act in preventing obesity [20], diabetes [21], and cancer [22].

As observed from the results of the phenolic contents, the levels of prenylflavonoids and hop acids in the four Saaz accessions considerably varied. In particular, 6-PN was either detected or undetected depending on the cultivated regions, and cohumulone content widely ranged from 9.42 (Saaz1) to 55.87 (Saaz2) µg/g, based on the spectrum of six hop cultivars. These results indicate that the levels of secondary metabolites, including flavonoids and phenolic compounds, were critically influenced by environmental conditions. Furthermore, previous studies have reported that the level of phenolics in the same cultivar can considerably change when cultivated under different environment regions [23,24].

### 2.3. Antioxidant Activities of Hop Strobile Extracts

The antioxidant capacity of the hop extracts was evaluated by the radical cation scavenging activity of their phenolic extracts (60% EtOH and water) and was measured using the DPPH and ABTS assay (Table 3). From the results of the DPPH assay, the IC_50_ range was practically similar between 60% EtOH and water extracts and varied from 124.25 to 342.29 ug/mL and from 192.41 to 342.60 ug/mL, respectively. However, in certain cultivars, the DPPH scavenging activity between the ethanol and water extracts differed significantly; for example, the antioxidant activity of Saaz3 and Cluster significantly decreased the levels of IC_50_ value in 60% EtOH extract, whereas the IC_50_ values significantly increased in the water extract. The ABTS scavenging assay also observed similar results; the IC_50_ values in Saaz3, Cluster, and El Dorado cultivars increased in water extract compared with that in 60% EtOH extract. Based on these results, few oxygen-sensitive antioxidants may be cultivar-specific, which are destroyed when exposed to high temperature during the boiled-water extraction process of hop strobile. In this regard, previous studies have reported conflicting results, although the radical scavenging assays used were different. Krofta et al. [25] reported that the high temperature treatment for pelletizing did not significantly affect the antioxidant status using DPPH assay, whereas Kowalczyk et al. [12] compared the data between hydroalcoholic and water extracts (tested using ABTS scavenging assay data) and suggested that the aqua-soluble primary antioxidants could be lost at high temperature during water extraction conditions. However, we additionally observed various patterns in both DPPH and ABTS assays; the IC_50_ values significantly increased (e.g., Saaz3, Cluster) and decreased (e.g., Saaz4, Calypso) in water extract, while the values were practically similar between 60% EtOH and water extracts (e.g., Saaz2 and Cascade). Therefore, we propose that the hops extract contains various aqua-soluble antioxidants, but these unknown compounds may be differentially accumulated in hop strobile, depending on the environmental conditions or hop cultivars.

Comparing the antioxidant capacity among the six hop cultivars based on the IC_50_ values, the El Dorado cultivar exhibited the highest antioxidant activity for both DPPH and ABTS assay (Table 3). Furthermore, El Dorado was recently cultivated in 2010 (USA), and comparable information on the antioxidant activity level for this cultivar is lacking. Therefore, these results could provide a novel dataset on antioxidant properties in hops breeding.

**Figure 3:**
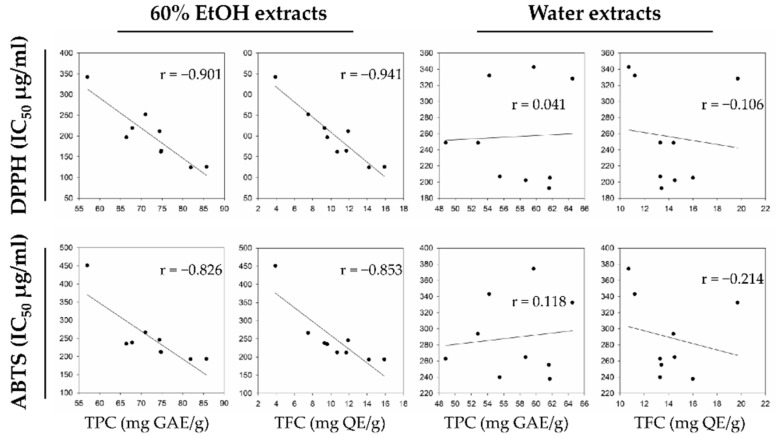
Scatter plot with Pearson correlation of relationship between antioxidant activities and phenolic compounds.

**Figure 4:**
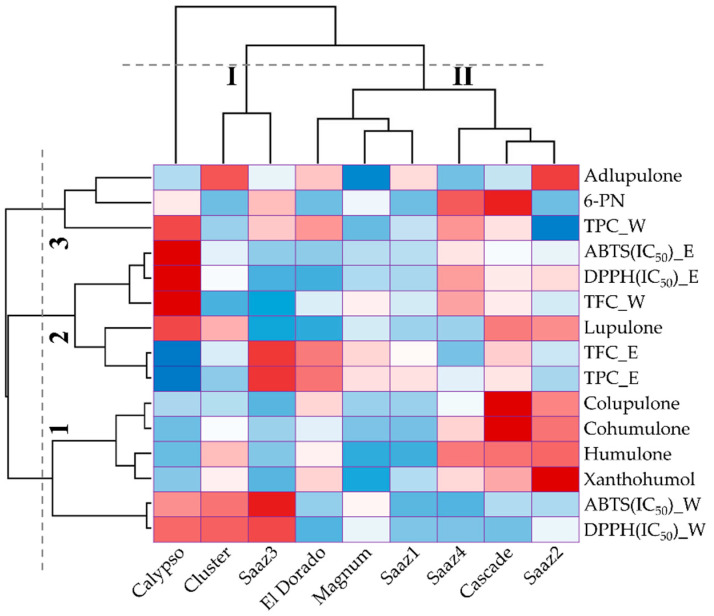
Hierarchical cluster analysis of nine different hop cultivars according to their phenolic levels and antioxidant activities.

In previous studies, antioxidant activity estimated by DPPH and ABTS assay has been correlated with TPC and TFC levels [8,12,26], mainly due to the redox properties of phenolic compounds [27]. To assess the relationship between the phenolic content in the hop strobile extracts and the antioxidant activities (DPPH and ABTS), we performed Pearson correlation analysis (Figure 3) and hierarchical clustering (Figure 4). In the 60% EtOH extracts, the DPPH scavenging activity (IC_50_) significantly correlated with TPC (−0.901) and TFC (−0.941). In addition, the ABTS activity (IC_50_) significantly correlated with TPC (−0.826) and TFC (−0.853) levels. In contrast, the antioxidant activities in water extracts were not significantly correlated with the phenolic content, because certain cultivars did not observe any correlation between phenolic compounds and antioxidant activities (e.g., Saaz3 and Calypso).

Hierarchical cluster analysis classified the accessions and measurements according to their phenolic content and antioxidant activities. The hop cultivars were divided into two clusters, among which one was independent; the Calypso cultivar was exhibited independently because of higher levels of DPPH and ABTS IC_50_ values. The first cluster contained the antioxidant activities that varied considerably due to the extraction solvents, and the second cluster was based on the status of the phenolic compounds and antioxidant properties. The measurements were classified into three groups: group1 practically contained prenylflavonoids and hop acids, group2 contained antioxidant activity with TPC and TFC, and group3 contained adlupulone, 6-PN, and water-extracted TPC (Figure 4). These results, as mentioned earlier, confirmed that the antioxidant activity was strongly associated with the levels of the TPC and TFC content.

Overall, the characteristics of antioxidant activities and composition of phenolic compounds differed in the Calypso cultivar compared with that of the other cultivars, and the antioxidant activities were associated with TPC and TFC in ethanol extract. The ABTS and DPPH activity in water extract exhibited marginal association with hop acids. However, the hop acids were extracted by different solvents; hence, verification using the same extraction solvent in further study is of utmost necessity.

## 3. Materials and Methods

### 3.1. Plant Material and Extraction

Nine accessions were used in this study. These included, Saaz (from four different cultivated regions), Calypso, Cascade, Cluster, El Dorado, and Magnum cultivars. The strobile of the hop samples were collected from Buan (Korea) and that of Saaz cultivar were collected from the respective four different regions, as described in Table 4. The strobile of hop plant were harvested and freeze-dried. Each sample (10 g) was extracted for 24 h with a mixture of 50 mL boiled-water and 60% ethanol. Sample extracts were subsequently used for estimating the total polyphenol content (TPC) and total flavonoid content (TFC).

### 3.2. Total Phenolic and Flavonoid Contents

The TPC was determined by the Folin–Ciocalteau colorimetric method [28]. The absorbance was measured at 760 nm using a UV-spectrophotometer (UV-1800, Shimazuda, Kyoto, Japan). TPC was calculated using a calibration curve with gallic acid as standard. The TFC in different strobile samples of hop cultivars was determined using the method proposed by AR Almeida et al. [7]. The absorbance was measured at 510 nm and TFC was calculated using a calibration curve of quercetin equivalents.

### 3.3. LC-MS Analysis of Prenylflavonoid Compounds

Powdered samples (each 1 g) from dried strobile of each hop cultivar were extracted with 1% formic acid in methanol (*v*/*v*, 20 mL) and ultrasonicated for 10 min [29]. Each extracted solution was filtered through a syringe filter (0.45 μm) for LC-MS analysis. Prenylflavonoid compounds were analyzed using an ultra-high performance liquid chromatography with a photodiode array detector (DAD; Agilent 1260 series; Agilent Technologies, Santa Clara, CA, USA) and quadrupole liquid chromatography/mass spectrometer (Agilent 6130; Agilent Technologies, Santa Clara, CA, USA) equipped with a SB-C18 column (150 × 4.6 mm i.d., 2.7 m particle size; Agilent Technologies), and a compatible C18 guard column (4 × 3 mm i.d.; 3 µm particle size; Phenomenex, Torrance, CA, USA). The mobile phase was composed of water (A, 0.05% formic acid) and acetonitrile (B, 0.05% formic acid). Prenylflavonoid compounds were separated using the following gradient: 0–5 min, 10–15% B; 5–10 min, 15–20% B; 10–25 min, 20–30% B; 25–35 min, 30–50% B; 35–40 min, 50–75% B; 40–55 min, 75–100% B; 55–60 min, 100–10% B, and were detected at 270 nm. Humulone and xanthohumol were identified by comparing them with the retention time of their commercial standards obtained from Sigma-Aldrich Co. (St. Louis, MO, USA) (UV spectrum). 6-Prenylnaringenin, cohumulone, colupulone, lupulone, and adlupulone were identified using the method described in a previous study [30] and by their UV-visible spectral characteristics.

### 3.4. Determination of DPPH Radical Scavenging Activity

2,2-diphenyl-1-picrylhydrazyl (DPPH) radical scavenging activity was measured as described previously [13]. Briefly, different concentrations of extract solutions were added to 0.15 mM DPPH in ethanol, and the reaction mixture was shaken vigorously. The amount of remaining DPPH radicals was determined using a plate reader (Benchmark Plus; Bio-Rad, Hercules, CA, USA) at 517 nm after 30 min.

### 3.5. ABTS Assay for Antioxidant Activity Evaluation

ABTS scavenging activity of different extracts of hop cone was determined as described previously [31]. The ABTS reagent was prepared by mixing 5 mL of 7 mM ABTS with 88 μL of 140 mM potassium persulfate. The mixture was then placed in dark at room temperature (23 ± 2 °C) for 16 h to allow free radical generation and was subsequently diluted with water (1:44, *v*/*v*). The scavenging activity was determined by mixing 100 μL ABTS reagent with 100 μL of sample in a 96-well microplate and was incubated at room temperature for 6 min. After incubation, the absorbance was measured at 734 nm. The results are expressed as ascorbic acid equivalent, which was used as a standard. The antioxidant activity was evaluated based on the IC_50_ value.

### 3.6. Statistical Analysis

Each experiment was performed in triplicate and all data are presented as the mean ± standard deviation (SD). The chemical analysis data subjected to analysis of variance (ANOVA) using a multiple comparisons method, Pearson correlation, and hierarchical clustering were analyzed using the SPSS version 12 statistical software package (SPSS Inc., Chicago, IL, USA).

## 4. Conclusions

In present study, the composition of phenolic compounds in the strobile extracts of six different hop cultivars were identified and their contributions toward the antioxidant activities were determined by DPPH and ABTS assay. In addition, we also investigated the changes in the phenolic levels and antioxidant activity in various solvent extracts and collected regions, which could provide additional evidence of antioxidant properties/efficiency in hop cultivars. The antioxidant activity was significantly associated with the levels of TPC and TFC in the ethanol extracts according to the statistical analysis. Moreover, this study is the first to report the antioxidant properties among various hop cultivars that includes the newly released hop (Calypso and El Dorado) cultivar. Especially, the ethanol extract of El Dorado cultivar exhibited higher levels of TPC content and antioxidant activities than other cultivars. The Calypso cultivar contained the highest water-soluble TFC and showed distinctly different properties of phenolic compound and antioxidant activity through hierarchical cluster analysis. These results can assist in exploring its potential as a valuable resource of medicinal crops for future antioxidant research. Further study is needed to demonstrate composition of the phenolic compounds from different extraction solvents and cultivated regions, which contribute to the antioxidant activity of hop strobiles.

## Figures and Tables

**Table 1:** Identification of prenylflavonoid compounds by LC-MS.

No.	Compound Names	tR (min)	Formula	Molecular Ions (m/z)
1	6-Prenylnaringenin	35.4	C_20_H_20_O_5_	341
2	Xanthohumol	38.4	C_21_H_22_O_5_	355
3	Cohumulone	45.4	C_20_H_28_O_5_	349
4	Humulone	46.9	C_21_H_30_O_5_	363
5	Colupulone	49.3	C_25_H_36_O_4_	401
6	Lupulone	50.5	C_26_H_38_O_4_	415
7	Adlupulone	50.7	C_26_H_38_O_4_	415

**Table 2:** Concentration of different prenylflavonoid compounds in hop accessions.

Cultivars	6-Prenyl Naringenin	Xanthohumol	Cohumulone	Humulone	Colupulone	Lupulone	Adlupulone
Calypso	12.42 d	20.36 e	8.44 g	65.48 d	36.78 d	52.10 a	5.36 cd
Cascade	29.78 a	37.88 b	77.87 a	188.27 a	73.00 a	46.96 ab	5.55 c
Cluster	ND	31.14 c	29.00 d	153.31 b	37.77 d	41.77 b	8.21 a
El Dorado	ND	33.81 bc	25.23 e	128.09 c	48.68 c	17.41 e	6.88 b
Magnum	8.97 e	15.06 f	10.17 g	50.44 e	35.41 d	29.40 c	3.58 e
Saaz1	ND	23.75 d	9.42 g	53.84 e	35.37 d	24.82 d	6.60 b
Saaz2	ND	53.60 a	55.87 b	193.25 a	57.13 b	45.13 ab	8.47 a
Saaz3	16.35 c	17.47 f	14.81 f	74.43 d	30.56 e	16.58 e	5.93 bc
Saaz4	24.96 b	33.33 bc	38.28 c	184.68 a	43.10 c	24.63 d	4.78 d

Values with different letters (a–f) are statistically different at *p* < 0.05 significant level (post hoc Duncan’s test); data represent the means ± SD (*n* = 3).

**Table 3:** Comparison of the antioxidant activities by 1,1-Diphenyl-2-picrylhydrazyl free radical scavenging assay (DPPH) and 2,2-Azino-bis-3-ethylbenzothiazoline-6-sulfonic acid assay (ABTS) of hop strobile extracts.

Cultivar	DPPH IC_50_ (µg/mL)		ABTS IC_50_ (µg/mL)	
	60% EtOH	Water	60% EtOH	Water
ASC *	4.20 ± 0.01		3.95 ± 0.00	
Calypso	342.29 ± 9.59 f	328.39 ± 7.91 c	451.29 ± 2.96 f	332.50 ± 1.56 e
Cascade	211.77 ± 13.25 cd	202.27 ± 2.41 a	246.08 ± 8.50 d	264.86 ± 2.34 c
Cluster	196.76 ± 3.69 c	332.07 ± 11.49 c	235.14 ± 3.14 c	343.11 ± 1.85 f
El Dorado	124.25 ± 3.10 a	192.41 ± 9.55 a	192.94 ± 4.47 a	255.34 ± 1.50 b
Magnum	164.46 ± 6.86 b	248.64 ± 3.28 b	212.13 ± 7.33 b	293.71 ± 0.37 d
Saaz1	161.85 ± 4.60 b	206.90 ± 6.87 a	212.89 ± 4.95 b	239.92 ± 1.06 a
Saaz2	219.41 ± 4.23 d	248.86 ± 9.50 b	238.42 ± 2.67 cd	262.87 ± 1.26 c
Saaz3	125.70 ± 0.93 a	342.60 ± 6.03 c	193.56 ± 1.44 a	374.47 ± 0.37 g
Saaz4	251.93 ± 4.89 e	205.31 ± 5.86 a	266.56 ± 3.03 e	237.90 ± 1.98 a

* Ascorbic acid (ASC): positive control in this study. Values with different letters (a–f) are statistically different at *p* < 0.05 significant level (post hoc Duncan’s test); data represent the means ± SD (*n* = 3).

**Table 4:** Nine different hop accessions used in this study.

No.	Variety Name	Cultural Regions (Latitude/Longitude)	Origin
1	Calypso	Buan (35°42′56″/126°43′33″)	USA
2	Cascade	Buan (35°42′56″/126°43′33″)	USA
3	Cluster	Buan (35°42′56″/126°43′33″)	USA
4	El Dorado	Buan (35°42′56″/126°43′33″)	USA
5	Magnum	Buan (35°42′56″/126°43′33″)	Germany
6	Saaz1	Buan (35°42′56″/126°43′33″)	Czech
7	Saaz2	Yeoungyang (36°30′55″/129°11′02″)	Czech
8	Saaz3	Yeoungdong (36°13′24″/127°42′28″)	Czech
9	Saaz4	Boryeong (36°11′16″/126°40′45″)	Czech

## Data Availability

Not applicable.
